# Prevalence of Type 2 Diabetes Mellitus in Hepatitis C Virus Infected Population: A Southeast Asian Study

**DOI:** 10.1155/2013/539361

**Published:** 2013-08-01

**Authors:** Muhammad Sadik Memon, Zain Islam Arain, Farukh Naz, Madiha Zaki, Suresh Kumar, Asif Ali Burney

**Affiliations:** Isra University Hospital, Hala Naka Road, Hyderabad, Pakistan

## Abstract

*Purpose*. The study was aimed to investigate the frequency of diabetes mellitus type 2 in patients infected with chronic hepatitis C virus and its association with cirrhosis. *Patients and Methods*. This prospective case series was conducted at Section of Gastroenterology and Hepatology, Isra University Hospital, Hyderabad, over a period of 4 months from June 2009 to October 2009. Hepatitis C virus seropositive patients who were older than 18 years, diabetic or nondiabetic, were included. Basic demographic data collected by questionnaire and laboratory investigations including fasting blood glucose levels, serum cholesterol, and liver function tests were done. A logistic regression model was used to explore the association between diabetic and nondiabetic HCV seropositives and type 2 diabetes mellitus with cirrhosis. *Results*. A total of 361 patients with hepatitis C were analyzed; the prevalence of type 2 diabetes mellitus in HCV patients was 31.5%. Out of the total number of the participants, 58.4% (*n* = 211) were cirrhotics, while 41.6% (*n* = 150) were noncirrhotic HCV seropositives. In multivariate analysis, cirrhotic patients appeared significantly more likely (*P* = 0.01) to be diabetic as compared with noncirrhotic patients (OR = 2.005, 95% CI: 1.15, 3.43). *Conclusion*. Advancing age, increased weight, and HCV genotype 3 are independent predictors of type 2 diabetes in HCV seropositive patients, and there is a statistically significant association of cirrhosis observed with type 2 diabetes mellitus.

## 1. Introduction

Hepatitis C virus (HCV) has been identified as one of the leading causes of chronic liver disease with serious sequel as the end stage of cirrhosis and liver cancer [1]. According to recent statistics, the worldwide prevalence of HCV infection is ~3% and affects around more than 170 million people globally [2]. Chronic hepatitis C infection mainly affects liver but can be associated with various extrahepatic manifestations including cryoglobulinemia, sialadenitis, glomerulonephritis, and porphyria cutanea tarda [3, 4].

Diabetes mellitus is a chronic disease of metabolism causing abnormal glucose homeostasis [5]. More than 171 million people globally are affected by diabetes mellitus, and the figure is expected to rise up to 366 million by 2030 [6]. A systemic review and meta-analysis from South Asia by Jayawardena et al. (2012) showed burden of diabetes in Pakistan ranging from 3% to 7.2% in a general population [7]. Type 2 diabetes mellitus in South Asian, when comparing with European individuals, is 4- to 6-fold more prevalent [8]. 

Several studies reported that HCV infection may also contribute to the development of diabetes, and higher prevalence of type 2 diabetes mellitus has been observed in the developed world (2% to 9.4%) in patients with HCV infection than in those with other forms of chronic hepatitis [9–12]. This association between HCV infection and diabetes was for the first time made by Allison et al. in 1994 [13]. Since then, a number of observational studies have been published.

There are several organized factors which influence the development of diabetes among HCV-infected patients like age, sex, family history of diabetes, African-American race, and HIV coinfection [14, 15].

Insulin resistance (IR) and diabetes can develop at any stage of HCV infection. Multiple mechanisms have been accounted for insulin resistance and development of diabetes in patients with chronic hepatitis C. It promotes IR mainly through interfering with insulin signaling pathway in hepatocytes, increasing inflammatory response with production of cytokines such as TNF alpha and IL-6 and increasing oxidative stress [16, 17].

There are many studies in the past done on frequency of diabetes in HCV infection. One such study was conducted by Elhawary et al. (2011) [15] that shows 13.84% prevalence of type 2 diabetes mellitus in HCV seropositive patients and also linked association of cirrhosis with diabetes mellitus.

HCV infection and type 2 diabetes mellitus are two chronic conditions which contribute to a significant morbidity and mortality. The rationale behind this study is to find out the maximum number of type 2 diabetes in those patients who are infected with hepatitis C virus and also the relation between cirrhosis and type 2 diabetes mellitus seropositive patients; so in this way, we will establish a valid association between type 2 diabetes in HCV seropositive population.

## 2. Patients and Methods

This prospective study was conducted at Isra University Hospital (IUH), Section of Gastroenterology and Hepatology, Hyderabad. IUH is a 300-bedded private, tertiary care academic teaching hospital that largely serves the residents of Hyderabad (population 2 million) and the surrounding 6–8 districts of Sindh province. The study protocol was evaluated and approved by the hospital authorities, from where all the study participants were recruited, and the study was conducted in accordance with the Declaration of Helsinki Guidelines. All the individuals provided informed consent before their participation. 

## 3. Inclusion Criteria

HCV seropositive patients, with or without presence of liver cirrhosis and/or diabetes mellitus type 2, between the age of 18 and 75 years, both males and females visiting the outpatient department or admitted in Gastroenterology and Hepatology Ward, were recruited consecutively during a period of 4 months from June 2009 to October 2009. Demographic information and all relevant data, such as area of residence and past medical history, were collected by trained nurses in a predesigned structured questionnaire. 

## 4. Exclusion Criteria

Patients with liver cancer, on interferon therapy, having end stage renal disease or coexisting viral infection like hepatitis B surface antigen positive patients, and pregnant females were excluded from the research.

## 5. Sample Collection

Senior laboratory technologist drew 5 mL venous blood sample using a sterilized disposable syringe, and then the sample was used for the detection of hepatitis C virus by enzyme-linked immunosorbent assay (ELISA) and HCV genotyping determined by using polymerase chain reaction (PCR). Diagnosis of type 2 diabetes mellitus was done according to the American Diabetes Association guidelines (2013) [18]. Other laboratory investigations include complete blood picture, serum cholesterol, serum albumin, total bilirubin, prothrombin time, and serum creatinine levels. Liver cirrhosis was confirmed if the patient had previous recent reliable reports available or by using radiological investigations and liver biopsy where needed.

## 6. Statistical Analysis

For the comparison of participants of those with and without type 2 diabetes, Student's *t*-test was used for normality distributed continuous variables. Categorical variables were presented as frequency and percentage, and chi-square test was used for nominal categorical variables comparison. The prevalence of type 2 diabetes mellitus among seropositive patients was calculated and presented as percentage. Age was categorized in two groups (<40 years and ≥40 years); body mass index (BMI) was calculated by weight in kg/height meter squared and then categorized in two groups (<27 and ≥27). Multiple logistic regression model was introduced with the dependent variables such as age, gender, weight, BMI, family history of diabetes, and HCV genotype. Initially, in order to include important variables, factors having significance *P* < 0.25 in univariate analysis were included in the multivariate analysis. The final model was selected using a forward method and *P* ≤ 0.05. Data entry and analysis were done with Statistical Package for Social Sciences, version 16 (SPSS Inc, Chicago, IL, USA).

## 7. Results

The mean age of the study population (± standard deviation) was 46.1 ± 12.3 with a range of 18–75 years of age. Male and female proportions were almost equal in our study sample, 52.6% and 47.6%, respectively. The majority of the study populations were married (94.4%) as compared to the single ones (5.54%). Overall prevalence of type 2 diabetes mellitus among HCV seropositive patients was 31.5% (Table 1).

Statistically significant proportion of HCV seropositive patients with cirrhosis was ≥40 years old (*P* ≤ 0.001). Other significant factors in HCV seropositive cirrhotics were male proportion, area of residence, marital status, and type 2 diabetes mellitus (*P* < 0.05) (Table 1).

Table 2 shows comparison of means between type 2 diabetics and nondiabetic seropositive patients. In univariate analysis, mean age difference (4.71 years), fasting blood sugar (71.2 mg/dL), albumin (0.22 mg/dL), serum cholesterol (70.4 mg/dL), and serum creatinine (0.24 mg/dL) were statistically significant (*P* < 0.05) as compared with nondiabetic HCV seropositive patients (Table 2). 

On multivariate binominal analysis, age ≥40 years, weight ≥70 kg, family history of diabetes, and HCV genotype 3 were significantly more likely to be diabetic (*P* < 0.05). Likelihood of being type 2 diabetic in HCV seropositive cirrhotic patients was significant in univariate analysis (*P* = 0.001), and in multivariate analysis, this association was observed two times higher as compared with noncirrhotic patients (OR = 2.005, 95% CI: 1.15, 3.43) (Table 3).

## 8. Discussion

We observed the prevalence of type 2 diabetes as 31.5% (114 cases) out of 361 hepatitis C seropositive patients. When we compared the prevalence of type 2 diabetes in HCV-infected population and noninfected population, a twice higher rate of type 2 diabetes was observed in our study as compared to noninfected population [19]. White et al. (2008) [20] analyzed 34 eligible retrospective and prospective studies, showing a significant risk of type 2 diabetes in HCV-infected group as compared to non-HCV-infected control group. On the other hand, recent data also suggest three times higher prevalence of type 2 diabetes in HCV seropositive patients [21]. In this way, data from the previous literature and from our study show a strong association between HCV and type 2 diabetes. Several reasons can explain the association of type 2 diabetes with HCV. One of the explanations is that the pathophysiology of HCV-associated type 2 diabetes mellitus consists of a defect in insulin secretion, increased hepatic tumor necrosis factor alpha, excessive hepatic glucose production, and insulin resistance, because the core-encoding region of HCV is sufficient to induce insulin resistance by the previously defined mechanism via either direct or indirect way [22]. Secondly, a major contribution of already present risk factors of diabetes such as positive family history and advancing age also plays an important role among HCV infected persons [23, 24].

Advancing age (≥40 years) in HCV seropositive patients was significantly associated with type 2 diabetes mellitus as compared to younger age group (≥18 to 40 years) on both univariate and multivariate analyses. The finding also agrees with another study conducted earlier on Mexican population [25].

It was observed in our study that positive cases of type 2 diabetes with respect to gender, education level, and marital status had insignificant relation. A study conducted by Elhawary et al., 2011, also revealed the same findings as in our study [15]; this could be because of the same number of patients attending the hospital, but this insignificant association between seropositive patients cannot be neglected, as in the general population, type 2 diabetes was more prevalent in males than in females [26, 27]; this relationship should be evaluated further on larger scale studies.

Fasting blood glucose levels and total serum cholesterol levels were significantly observed when diabetic compared with nondiabetic HCV seropositive patients. Substantial evidence from the previous literature shows significant association between these laboratory findings [15, 21]. Additionally, patients with HCV and type 2 diabetes may involve kidneys and patients may present with acute renal failure during its later stages [4] which can be observed through significant association of raised serum creatinine in diabetic HCV positive patients [28]. In our study, an increase in mean serum creatinine was significantly observed in HCV patients with type 2 diabetes as compared to HCV patients who were not diabetic. 

The most common genotype in our study was genotype 3 which constituted >85% of the whole sample. Previous literatures from Pakistan favor our findings [29–32]; as HCV genotype 3 is more common in Pakistan [33]. Controversies regarding the presence of a specific genotype in HCV seropositive diabetic patient are still a part of scientific debate, and larger scale studies are required to find a conclusion regarding which genotype is more specifically affecting diabetic HCV seropositive patients [34–36]. However, our study observed that the HCV seropositive population with genotype 3 was significantly associated with type 2 diabetes mellitus. This significant association could be due to genotype 3 in our study comprises of more than third portion of whole HCV diabetic population. 

Our study also observed that odds of being type 2 diabetics among HCV-infected patients with cirrhosis was doubled (OR = 2.005) as compared to noncirrhotic HCV seropositive patients. Importantly, studies published in other areas also describe similar trends, in which type 2 diabetes mellitus is more frequently observed in patients with cirrhosis as compared to noncirrhotics, and it ranges from 19.5% to 50% [37–40]. This relationship proves that with advancing liver disease there will be increased susceptibility of HCV seropositive patients to develop type 2 diabetes mellitus.

## 9. Study Limitations

There are certain limitations of this study. The most important limitations in this study are small sample size, single-centered-hospital-based study, and only included HCV seropositives which may be unable to reflect the actual incidence of type 2 diabetes in HCV seropositives.

## 10. Conclusion and Recommendation

This study concludes that there is a strong association of type 2 diabetes mellitus with HCV infection. Odds of development of type 2 diabetes increases by twofolds if an HCV patient has cirrhosis. Advancing age, increased weight, and HCV genotype 3 are independent predictors of type 2 diabetes in HCV seropositive patients. It is necessary to screen and control earlier for the presence of type 2 diabetes mellitus and also rule out HCV infection among diabetic populations which is rarely done on population-based studies.

## Figures and Tables

**Table 1:** Demographic and clinical characteristics of study participants.

Characteristic	HCV cases *N* = 361 *N* (%)	Cirrhotic *N* = 211 *N* (%)	Noncirrhotic *N* = 150 *N* (%)	*P* value
Age—years (mean ± SD)	46.1 ± 12.3	50.6 ± 11.6	39.8 ± 10.5	<0.001*
Range	18–75	18–75	18–75	—
≥18–40	127 (35.1)	44 (20.8)	83 (55.3)	<0.001*
≥40	234 (64.8)	167 (79.1)	67 (44.6)	
Gender				
Male	190 (52.6)	124 (58.7)	66 (44)	0.006*
Female	171 (47.3)	87 (41.2)	84 (56)	
Residence				
Urban	171 (47.3)	88 (41.7)	83 (55.3)	0.01*
Rural	190 (52.6)	123 (58.2)	67 (44.6)	
Education				
<5 years	234 (64.8)	124 (58.7)	92 (61.3)	0.24
≥5 years	127 (35.1)	87 (41.2)	58 (38.6)	
Marital status				
Single	20 (5.54)	7 (3.31)	13 (8.66)	0.02*
Married	341 (94.4)	204 (96.6)	137 (91.3)	
Weight—kg				
<70	232 (64.2)	135 (63.9)	97 (64.6)	0.89
≥70	129 (35.7)	76 (36.0)	53 (35.3)	
BMI—kg/m^2^				
<27	176 (48.7)	108 (51.1)	68 (45.3)	0.27
≥27	185 (51.2)	103 (48.8)	82 (54.6)	
Type2 DM				
Yes	114 (31.5)	81 (38.3)	33 (22)	0.001*
No	247 (68.5)	130 (61.6)	117 (78)	
Family history of DM				
Yes	109 (30.1)	58 (27.4)	51 (34)	0.18
No	252 (69.8)	153 (72.5)	99 (66)	
Viral genotypes				
Genotype 1	33 (9.14)	31 (14.6)	17 (11.3)	0.73
Genotype 2	3 (0.83)	2 (0.94)	1 (0.66)	
Genotype 3	311 (86.1)	171 (81.0)	125 (83.3)	
Genotype 4	14 (3.87)	7 (3.31)	7 (4.66)	

*Statistically significant *P* value < 0.05.

DM: diabetes mellitus, BMI: body mass index.

**Table 2:** Unpaired *t*-test for the mean differences between diabetic and nondiabetic HCV seropositive patients.

Parameters	HCV cases		Mean difference type 2 DM/non diabetic	*P* value	95% CI
	Diabetic type 2 *N* = 114 Mean ± SD	Non Diabetic *N* = 247 Mean ± SD			
Age—years	49.3 ± 11.0	44.6 ± 12.6	4.71	0.001*	1.38, 7.42
FBS—mg/dL	157.1 ± 47.4	85.8 ± 12.2	71.2	<0.001*	64.9, 77.6
BMI—kg/m^2^	27.7 ± 5.48	26.8 ± 5.31	0.99	0.1	−0.20, 2.18
T. Bilirubin—mg/dL	1.67 ± 3.51	1.86 ± 3.83	0.19	0.65	−0.64, 1.02
Prothrombin time	15.9 ± 4.77	15.4 ± 5.22	0.53	0.35	−0.59, 1.66
Albumin—mg/dL	3.22 ± 0.71	3.44 ± 0.68	0.22	0.004*	0.07, 0.37
Cholesterol—mg/dL	200.6 ± 22.4	130.2 ± 16.8	70.4	<0.001*	66.2, 74.6
Creatinine—mg/dL	1.07 ± 0.88	0.82 ± 0.48	0.24	0.001*	0.10, 0.38
Weight—kg	67.8 ± 15.4	64.7 ± 14.0	3.14	0.05	−0.07, 6.36

*Statistically significant *P* value < 0.05.

FBS: Fasting blood sugar, BMI: Body mass index, CI: Confidence interval.

**Table 3:** Risk factor distribution between diabetic and non diabetic HCV seropositive subjects.

Risk	Univariate analysis			Multivariate analysis		
	Diabetic type 2 (*N* = 114) *N* (%)	Nondiabetic (*N* = 247) *N* (%)	*P* value	Adjusted OR	95% CI	*P* value
Age—years						
18–40	27 (23.7)	100 (40.5)	0.002*	1	1.34, 4.39	0.003*
≥40	87 (76.3)	147 (59.5)		2.42		
Gender						
Female	51 (44.7)	120 (48.6)	0.49	1	0.57, 1.58	0.84
Male	63 (55.3)	127 (51.4)		0.95		
Weight—kg						
<70	65 (57.0)	167 (67.6)	0.05	1	1.23, 4.93	0.01*
≥70	49 (42.9)	80 (32.4)		2.47		
BMI—kg/m^2^						
<27	55 (48.3)	121 (48.9)	0.89	1	0.29, 1.10	0.09
≥27	59 (51.7)	126 (51.0)		0.56		
Family history of diabetes						
No	60 (52.6)	192 (77.7)	<0.001*	1	0.15, 0.43	<0.001*
Yes	55 (48.4)	55 (22.3)		0.25		
HCV genotype						
Other genotypes^†^	6 (5.26)	44 (17.8)	0.001*	1	1.57, 10.13	0.003*
Genotype 3	108 (94.7)	203 (82.2)		3.99		
Disease status						
Noncirrhotic	33 (28.9)	117 (47.3)	0.001*	1	1.15, 3.43	0.01*
Cirrhotic	81 (71.0)	130 (52.7)		2.005		

*Statistically significant *P* value < 0.05.

OR: odds ratio; CI: confidence interval; BMI: body mass index.

^†^Other genotypes: genotype 1, genotype 2, and genotype 4.
